# Stress and sleep deprivation-related biomarkers in saliva in patients with retinitis pigmentosa

**DOI:** 10.1371/journal.pone.0304261

**Published:** 2024-06-13

**Authors:** Milagros Mateos-Olivares, Salvador Pastor-Idoate, Javier Martín-Vallejo, Cristina García-Vazquez, José Carlos Pastor, Ricardo Usategui-Martín, Eva María Sobas

**Affiliations:** 1 Moorfields Eye Hospital NHS Foundation Trust, London, United Kingdom; 2 Department of Ophthalmology, Clinical University Hospital of Valladolid, Valladolid, Spain; 3 Institute of Applied Ophthalmobiology (IOBA), University of Valladolid, Valladolid, Spain; 4 Networks of Cooperative Research oriented to Health Results (RICORS), Carlos III Health Institute, Madrid, Spain; 5 Department of Statistics, Institute of Biomedical Research of Salamanca (IBSAL), University of Salamanca, Salamanca, Spain; 6 Faculty of Medicine, Department of Cell Biology, Genetics, Histology and Pharmacology, University of Valladolid, Valladolid, Spain; 7 Faculty of Nursing, University of Valladolid, Valladolid, Spain; Federal University of Rio Grande do Sul: Universidade Federal do Rio Grande do Sul, BRAZIL

## Abstract

**Purpose:**

Patients with Retinitis Pigmentosa (RP) commonly experience sleep-related issues and are susceptible to stress. Moreover, variatiaons in their vision are often linked to anxiety, stress and drowsiness, indicating that stress and sleep deprivation lead to a decline in vision, and vision improves when both are mitigated. The objective of this study was to investigate the utility of salivary biomarkers as biochemical indicators of anxiety and sleep deprivation in RP patients.

**Methods:**

Seventy-eight RP patients and 34 healthy controls were included in this observational study. Anxiety and sleep-quality questionnaires, a complete ophthalmological exam for severity grading and, the collection of salivary samples from participants were assessed for participants. The activity of biomarkers was estimated by ELISA, and statistical analysis was performed to determine associations between the parameters. Associations between underlying psychological factors, grade of disease severity, and biomarkers activity were also examined.

**Results:**

Fifty-two (67%) of patients had a severe RP, and 26 (33%) had a mild-moderate grade. Fifty-eight (58,9%) patients reported severe levels of anxiety and 18 (23.,1%) a high level. Forty-six (59%) patients obtained pathological values in sleep-quality questionaries and 43 (55.1%) in sleepiness.

Patients with RP exhibited significant differences in testosterone, cortisol, sTNFαRII, sIgA and melatonin as compared to controls and patients with a mild-moderate and advanced stage of disease showed greater differences. In covariate analysis, patients with a severe anxiety level also showed greater differences in mean salivary cortisol, sTNFαRII and melatonin and male patients showed lower IgA levels than female.

**Conclusions:**

The present findings suggest that salivary biomarkers could be suitable non-invasive biochemical markers for the objective assessment of sleep deprivation and anxiety in RP patients. Further research is needed to characterize the effects of untreated negative psychological states and sleep deprivation on increased variability of vision and disease progression, if any.

## Introduction

Retinitis pigmentosa (RP) is a group of inherited retinal disorders where the progressive loss of photoreceptors (rods in the first stage and later rods and cones) results in severe visual impairment and irreversible blindness [1–3]. Despite RP is considered a rare disease, its prevalence is 1 in 3000 to 1 in 5000, being the most common inherited retinal dystrophy worldwide [1,4,5].

In RP patients, the slowly progressive worsening in vision, for which there is no currently available cure, often has serious implications in their everyday life activities. Because vision loss is irreversible and progressive, patients can develop negative psychological states such as anxiety, mental stress, depression, circadian rhythm disturbances, and sleep-related problems which are interrelated [6,7]. It has been reported that patients with progressive visual loss are more vulnerable to sleep disorders and have higher susceptibility towards stress and anxiety [8–10]. While prolonged mental stress and sleep-related problems can be a consequence of vision loss, both may also aggravate the situation. In addition, variations in their vision are often related to mental stress, stating that hassle causes a decrease in vision and sleep-related problems, and both improve when the stressor disappears [11].

The diagnosis of stress, anxiety, or sleep disorders, which are closely related to each other, is usually based on patient observation, medical history, physical exam, and interview with the patient and their family. Additionally, the so-called diagnostic tests (e.g., State-Trait Anxiety Index (STAI), Pittsburg Scale Quality Index (PSQI) or Epworth Sleepiness Scale (ESS) can assist this task [12–16]. However, these self-reported measures provide subjective information concerning anxiety, and sleep quality. Thus, their diagnosis relies on other clinical parameters and overlapping symptoms, hindering their diagnosis and effective treatment [16]. To date, there are no specific laboratory tests for the appraisal of stress, anxiety, or sleep disorders in RP patients, nor the assessment of the severity of the disease or therapy’s effectiveness.

Considerable efforts have been previously made to establish valid biomarkers for the assessment of anxiety, stress and sleep-related problems. To explore such biomarkers, saliva seems to be one of the commonest recommended and suitable mediums, due to the ease of its collection not causing discomfort to the patients [17]. Among such biomarkers, salivary alpha-amylase (sAA), cortisol, melatonin, testosterone, soluble fraction of receptor II of Tumor Necrosis Factor-α (sTNFαRII), and secretory Immunoglobulin-A (sIgA) have been studied widely and recommended as markers of activity of the sympathetic adrenomedullary system (SAS)(catecholamines), the hypothalamic-pituitary-adrenal (HPA) (hormones) and sleep deprivation [18–23]. The SAS and HPA activity are also known to increase due to sleep disorders [24] and melatonin has a certain inhibitory effect on the stress response and can alleviate the damage caused by acute and chronic stress by regulating the SAS and HPA axis [25].

The use of salivary stress and sleep deprivation-related biomarker concentrations (sSSBC) as biochemical markers in ophthalmic diseases has been previously reported in recent studies supporting the idea that sSSBC may be a useful tool to monitor disease activity or progression [26–29]. However, to the best of our knowledge, sSSBC has not been previously studied in individuals with RP.

In the present study, we evaluated different stress and sleep deprivation-related biomarkers in a cohort of patients with RP. To investigate the suspected relationship between sleep-related problems, anxiety and stress and, episodes of vision loss associated with RP disease, patients were graded into severity disease groups, anxiety status and sleep quality were analyzed by STAI, PSQI and ESS questionnaires and patients´ salivary biomarkers activity data were compared to healthy controls from the general population.

## Materials and methods

### Study design and participants

In this observational pilot study, salivary stress, and sleep deprivation-related biomarkers, more concretely sAA, cortisol, melatonin, testosterone, sTNFαRII, and sIgA, were determined from saliva samples collected from RP patients and healthy controls in two visits at least > 72 hours apart.

In addition, RP patients were graded into severity disease groups, and their anxiety status and sleep quality were analyzed by STAI, PSQI and ESS questionnaires.

Seventy-eight participants were examined between 11^th^ January 2021 and 16^th^ December 2022 at the Department of Ophthalmology of Clinical University Hospital of Valladolid and the Institute of Applied Ophthalmobiology (IOBA), University of Valladolid, Valladolid, Spain.

RP patients were recruited from RECYL (Association of RP Patients from Castilla y León, Spain) and two study groups were formed, the RP group (n = 78) and a second group of healthy controls (n = 34). The control group (n = 34) was formed to obtain reference values for normal sAA, cortisol, melatonin, testosterone, sTNFαRII, and sIgA values and in healthy subjects without a history of retinal diseases.

The inclusion criteria in the RP group (n = 78) were (1) had RP diagnosis, (2) were aged from 18 to 60, and (3) were of European descent.

The exclusion criteria for all groups were: (1) presence of other retinal pathologies, (2) a history of ocular surgery at least 6 months before, (3) uveitis or glaucoma, (4) a history of severe mental illness (including delirium, psychosis, disorganized behavior, hallucinations or extreme mood disorders such as mania, depression or severe anxiety disorder), (5) patients with inflammatory or autoimmune diseases, (6) pregnancy or lactation, (7) hormonal treatment including oral contraceptives, (8) current treatment of psychotropic, anti-inflammatory, or analgesic drugs.

The study protocol was approved by the Medical Ethics Committee of the Clinical University Hospital of Valladolid (“CEIC/CEIm Área de Salud Valladolid Este”; PI-GR-18-938 PI 17–732). Written consent was obtained from each participant before examinations.

### Ophthalmological exam and degree of Retinitis Pigmentosa severity

RP severity grading has been previously described in detail in a previous publication of the group [30]. In brief, RP patients were subjected to a complete ophthalmological exam (visual acuity, fundoscopy, optical coherence tomography and visual field test) and graded into severity disease groups according to Smith et al. [31].

The RP patients were graded from mild to severe on a 0–4 point scale. Clinical assessment included an evaluation of 4 of the distinguishing clinical signs in RP. The presence of visually significant lens opacity, moderate or severe optic disc atrophy, bone spicules or macular pathology (epiretinal membrane or cystoid macular oedema) scored 1 point each. Ancillary tests consisted of best corrected visual acuity (BCVA) (LogMAR), mean deviation (MD) in automated perimetry (dB) and Spectral Domain-Optical Coherence Tomography (SD-OCT)-measured outer retinal thickness (μ) at the fovea.

As a result of the sum of clinical assessment and ancillary test scores, the total severity grade (TSG) variable was calculated. It ranged from 0 to 16, considering punctuations between 0–3 a mild disease grade, 4–7 moderate and 8–16 severe. When total severity grade was stablished in our patients, most of them resulted in an advanced stage of disease and it was decided to stratify patients into only two groups mild-moderate named grade 2 and severe disease, grade 3 [31].

#### Anxiety status and sleep quality assessment

Questionnaires were distributed directly and self-answered, when the subjects had reading ability, after pertinent explanations. In case they could not read, the collaborating investigators read it to them and wrote down the answers.

#### The State-Trait Anxiety Inventory (STAI) questionnaire

STAI questionnaire was used to characterize the anxiety level perceived by the participants. The questionnaire was completed twice, at the first and second visits. Those visits were separated for a week. The results of STAI were categorized into 4 groups: 1. Not anxious (0–19), 2. Mild grade (20–28), 3. High grade (29–39), 4. Severe grade (>40) [12–14].

#### Pittsburgh Sleep Quality Index (PSQI) questionnaire

PSQI questionnaire was used to assess sleep quality and disturbances. The PSQI was administered in visit 1. The PSQI includes a scoring key for calculating a patient’s seven sub-scores, each of which can range from 0 to 3. The seven component scores are then summed to yield a global PSQI score, which has a range of 0–21. Score > 5 yielded distinguished good and poor sleepers [32].

#### Epworth Sleepiness Scale (ESS) scale

Sleepiness level among RP patients was measured on ESS. The ESS was also administered in visit 1. The ESS score (the sum of 8 item scores, 0–3) can range from 0 to 24. EES score of 10 or more was indicated as excessive sleepiness during common daily activities [16].

#### Data and salivary sample collection and measurement

Relevant sociodemographic variables (age, smoking status, body mass index, employment status, physical exercise, or sex) and ocular and medical histories were recorded. Sample collection was performed in a controlled ventilation chamber at the IOBA [33] and then we further analyzed salivary samples from all participants.

Collections were performed in a clinical setting from each subject in two visits between 9 a.m. and 12 p.m. to minimize potential errors associated with the diurnal variations in neuroendocrine parameters. Two single samples of saliva were collected from each subject, with an interval goal of 72 hours. Sample collection method and analysis have been described in previous publications of the group [34]. In brief, subjects were individually instructed on how to perform saliva collection using the passive secretion method over a 5-minute period into a collection tube. Collection of at least 1 mL was required. If the 5-mL collection tube was filled before 5 min, the amount of elapsed time was recorded. Visible blood contamination required that the sample be discarded, and after a 10-minute wait, a new sample was collected. All samples were collected in the same room, and temperature and humidity were recorded. Then, samples were frozen at -20°C and stored until they were analyzed.

In addition, the potential effect of the female cycle on the biomarkers’ levels was taken into consideration, assessing saliva concentration of biomarkers in female subjects according to the menstrual cycle phase: follicular phase (from 1 to 14 days), luteal phase (from 15 to 28 day) and hemorrhagic phase [35,36].

The following putative salivary indicators of stress and sleep deprivation were assayed by enzyme-linked immunosorbent assay (ELISA) following the manufacture instructions: sAA (Salimetrics™, State College, PA, USA), cortisol (DRG Instruments GmbH, Marburg, Germany), melatonine (Melatonin direct Saliva ELISA, IBL International GmbH, Hamburg, Germany), testosterone (DRG Instruments GmbH), sTNFαRII (Quantikine, Human sTNF RII/TNFRSF1B Immunoassay, R&D Systems, Minneapolis, MN, USA), and, sIgA (The Binding Site Group, Ltd., Birmingham, UK).

### Statistical analysis

SPSS Statistic version 22.0 was used for statistical analysis (SPSS, Chicago, IL, USA), and statistical significance was set at p<0.05. Biomarker variation and intersession reliability were assessed with the intraclass correlation coefficient (ICC). Data were presented as mean and standard deviation (SD) unless mentioned otherwise. Analysis of Variance (ANOVA) and paired *t* test were used for comparison of data in different and related groups, respectively. Pearson correlation was used to assess associations.

Chi-squared test was used to compare the categorical variables and odd ratios (ORs) and 95% confidence intervals (95%CIs) were estimated using an unconditional logistic regression model. The adjusted mean values were back-transformed, and 95% CIs were determined.

Salivary cortisol, melatonin and testosterone concentrations were logarithmically transformed as hormone values were not normally distributed. In addition, analyses of salivary biomarkers were stratified according to age, grade of severity, anxiety status, and gender.

The Shapiro-Wilk and Kolmogorov-Smirnov tests were employed to evaluate the distribution of the data both at baseline and after logarithmic transformation. Our results from the Shapiro-Wilk and Kolmogorov-Smirnov tests for the baseline data yielded p-values of 0.01 and 0.04, respectively, clearly indicating a deviation from normality. However, the results after the logarithmic transformation, with a p-value >0.05 for both tests, suggest a marginal fit to a normal distribution.

A linear regression model was used to describe the relationships among the variables. To meet its application requirements, potential confounders such as grade of severity of RP disease, anxiety status, sex, and age (since hormone production tends to vary with aging [37]) were corrected. In addition, post hoc sensitivity analysis excluding outliers was used for comparisons.

## Results

### Baseline characteristics, anxiety status and sleep quality assessment

Baseline characteristics and severity grading of RP patients and controls are summarized in Table 1 [30,34]. In brief, 78 patients with RP (47 (60.3%) males) and 34 healthy controls (11 (32.35%) males) were included. The mean age in the patients’ group waswas 51.59 ± 13.40 years old, and 34.26 ± 2.26 years old in the control group. Patients with RP were 17 years older than controls (*p* = 0.001). Controls were also ophthalmologically assessed and none of them showed anything pathological or had a familiar history of RP.

**Table 1:** Baseline characteristics, anxiety status and sleep quality assessment.

		RP patients; *n* = 78		Controls; *n* = 34		p-value
Age, yrs (mean, SD)		51.59 (13.40)		34.26 (2.27)		0.001 (*t*)
Sex, male/female		47/31		11/23		0.006 (*x*^*2*^)
Grading severity		52 (67%) severe 26 (33%) mild-moderate		-		-
Anxiety status	not anxious (0–19)	2 (2.55%)		34 (100%)		<0.001 (*x*^*2*^)
	mild grade (20–28)	-		-		
	high grade (29–39)	18 (23.1%)		-		
	severe grade (>40)	58 (58.97%)		-		
Sleep quality	normal	32 (41.02%)		34 (100%)		<0.001 (*x*^*2*^)
	poor sleepers	46 (58.98%)		-		
Sleepiness	normal	35 (44.87%)		34 (100%)		<0.001 (*x*^*2*^)
	mild	32 (41.02%)		-		
	moderate	11 (14.2%)		-		
Duration of RP disease, yrs (mean, range)		25.4 (2–50)		-		-
Smoking or drugs (No/Yes)		76/2		30/4		0.067 (*x*^*2*^)
BMI: (kg/m^2^) (mean, SD)		23.34 (2.94)		21.51 (4.97)		0.017 (*t*)
Employment status	Student	3		-		-
	Employed	15		34		<0.001 (*x*^*2*^)
	Unemployed	42		-		-
	Retired	18		-		-
Regular physical exercise, No/ Yes		56/22		20/14		0.192 (*x*^*2*^)
Menstrual cycle phase	1^st^ and 2^nd^ sample	1^st^	2^nd^	1^st^	2^nd^	-
	Follicular phase	12	13	14	16	>0.05* (*x*^*2*^)
	Luteal phase	2	1	6	4	>0.05*(*x*^*2*^)
	Hemorrhagic phase	1	1	3	3	>0.05*(*x*^*2*^)
Perimenopause or menopause status		16		-		-

Data are presented as standard deviation (SD), mean (range) or as numbers. RP: Retinitis Pigmentosa, yrs: Years. BMI: Body mass index.

* Both samples. p values were calculated using the Chi-squared (*x*^*2*^) and paired *t* test (*t*).

Regarding the RP grade, 52 (67%) of patients had a severe RP and 26 (33%) had a mild-moderate grade when they were evaluated and had been diagnosed with RP by an ophthalmologist for a mean of 25.4 years (range: 2–50) [30,34].

Most patients showed a high (“STAI-status 29–39”) or severe (“STAI-status >40”) anxiety status according to the questionnaire’s results with 23.1% (n = 18) and 74.35% (46 patients) of RP patients respectively. Patients did not show significant differences in anxiety scores according to their disease grade [30,34].

Forty-six (59%) of RP patients (n = 32 severe RP and 14 mild-moderate RP) obtained pathological values in PSQI, and 43 RP patients (55.1%) (n = 28 and 15, respectively) showed scores >10 points in EES, without significant difference between groups [30,34].

### Baseline clinical characteristics of RP patients

Clinical appearance data has also been described in a previous publication [30]. Table 2 summarizes the baseline clinical characteristics of RP patients.

**Table 2:** Baseline clinical characteristics of RP patients.

		Severe RP; *n* = 52	Mild-moderate RP; *n* = 26	p-value
Visual acuity (mean, SD)		1.35 (0.46)	0.29 (0.30)	0.001 (*t*)
Clinical appearance	Significant lens opacity	13	4	0.396 (*x*^*2*^)
	Optic disc atrophy	17	2	0.023 (*x*^*2*^)
	Bone spicules	45	13	0.001 (*x*^*2*^)
	Macular pathology	6	1	0.414 (*x*^*2*^)
Perimetry dB (mean, range)		-30 (-33.4 to -22.5)	-23.1 (-31.3 to -5)	0.001 (*t*)
ORT at the fovea (mean, SD)		150.7 (23.48)	179.4 (17.18)	0.001 (*t*)

Data are presented as standard deviation (SD), mean (range) or numbers. RP: Retinitis Pigmentosa, dB: Decibel, ORT: Outer retinal thickness, p values were calculated using the Chi-squared (*x*^*2*^) and paired *t* test (*t*).

### Salivary stress and sleep deprivation-related biomarkers concentrations (sSSBC)

The collection of salivary samples succeeded in 75 patients. 3 samples were discarded from the final analysis due to blood visible contamination. 15 patients were under systemic treatments: levothyroxine, antihypertensive drugs, betahistine, and medications used for treating heartburn, and acid reflux. After reviewing the technical information on these treatments, we considered not to exclude these samples from the final analysis. Since there were significant age and sex differences between the two groups (RP patients and controls) sSSBC study was adjusted by age and sex.

**Fig 1:**
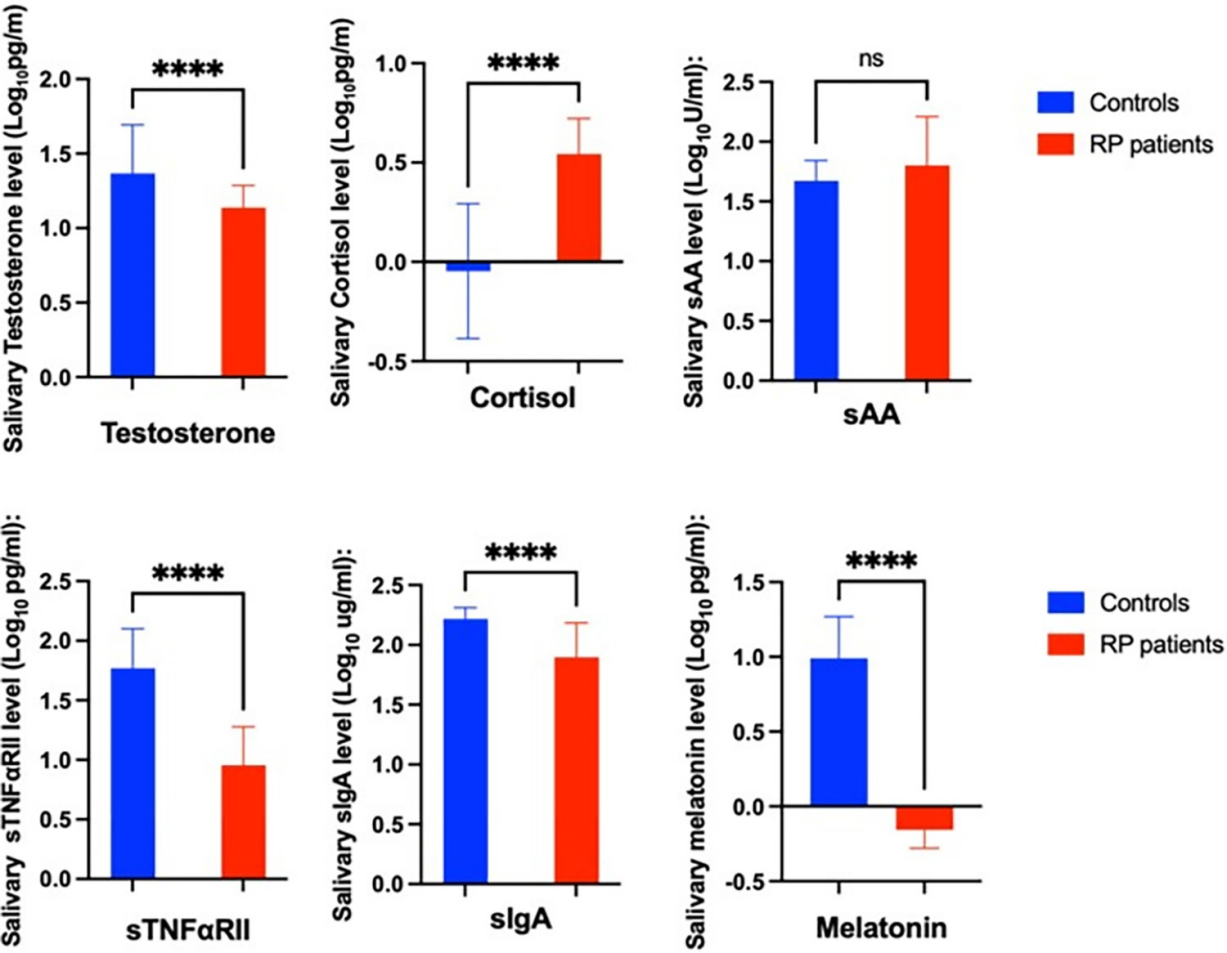
Mean ± SD Log-transformed sSSBC and differences between groups. Unpaired t-test results.

Salivary cortisol, melatonin and testosterone concentrations were logarithmically transformed as these hormone values were not normally distributed. Thus, logarithmic transformation was done in all salivary biomarkers for statistical analysis. Mean ± SD in testosterone (1.367 ± 0.324 Log_10_ pg/ml) (p < 0.001), cortisol (0.542 ± 0.178 Log_10_ pg/ml) (p < 0.001), sTNFαRII (0.955 ± 0.322 Log_10_ pg/ml) (p < 0.001), sIgA (1.896 ± 0.288 Log_10_ ug/ml) (p < 0.001), and melatonin (-0.155 ± 0.124 Log_10_ pg/ml) (p < 0.001) were significantly different compared to controls values (Fig 1). However, no significant differences were found in sAA concentrations between two groups (Fig 1).

**Fig 2:**
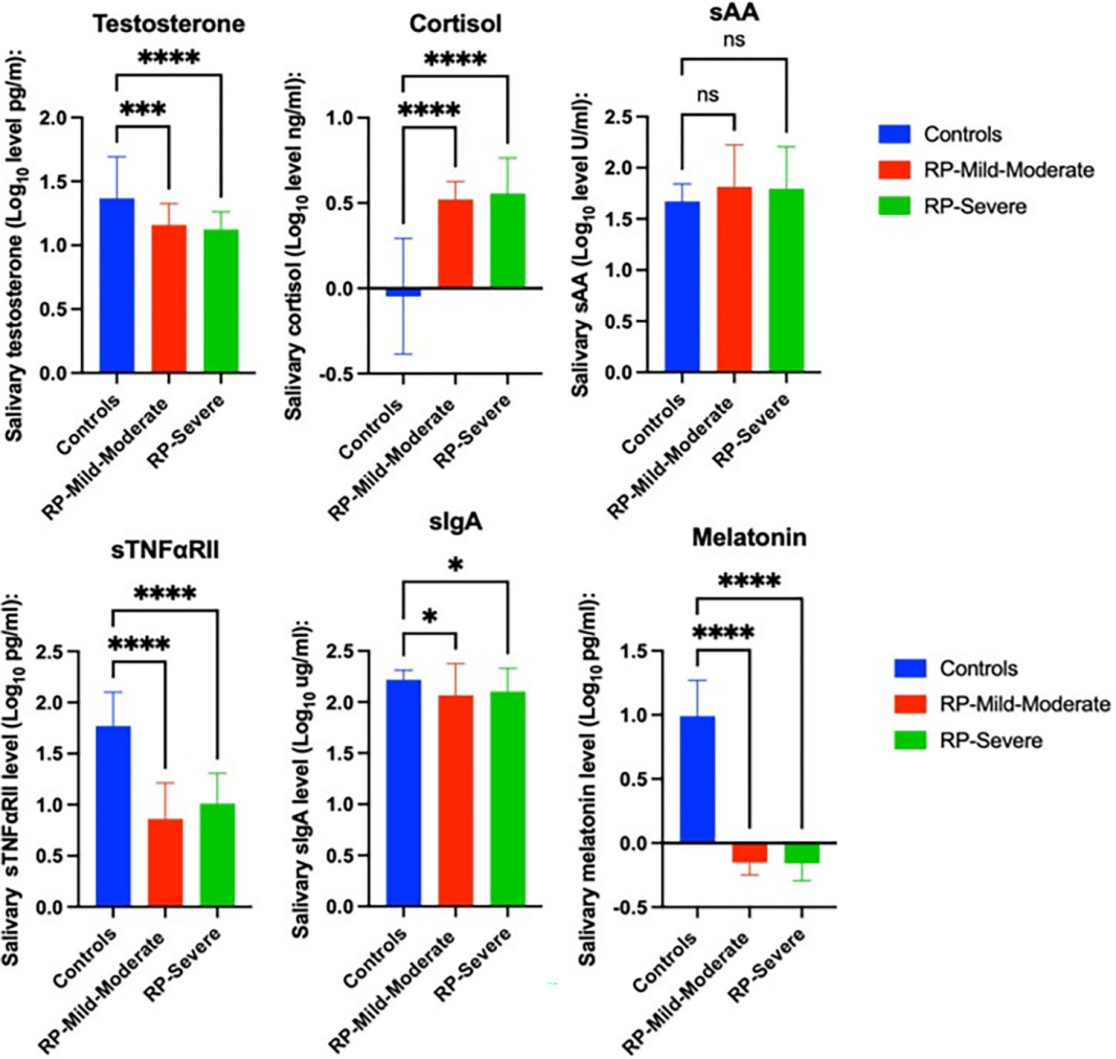
Mean ± SD Log-transformed sSSBC and differences between groups according to disease stage. ANOVA results.

Mean salivary testosterone (1.16 ± 0.165 Log_10_ pg/ml) (p < 0.01), (1.124 ± 0.138 Log_10_ pg/ml) (p < 0.001); cortisol (0.521 ± 0.104 Log_10_ pg/ml) (p < 0.001), (0.555 ± 0.209 Log_10_ pg/ml) (p < 0.001); sTNFαRII (0.861 ± 0.352 Log_10_ pg/ml) (p < 0.001), (1.009 ± 0.295 Log_10_ pg/ml) (p < 0.001); sIgA (2.066 ± 0.309 Log_10_ ug/ml) (p < 0.05), (2.104 ± 0.2261 Log_10_ ug/ml) (p < 0.05); and melatonin (-0.1511 ± 0.097 Log_10_ pg/ml) (p < 0.001), (-0.1573 ± 0.1376 Log_10_ pg/ml) (p < 0.001) concentrations were also significantly different in control than in RP patients with a mild-moderate and advanced stage of the disease (Fig 2). However, no differences were found between RP patients with a mild-moderate and advanced stage of the disease.

Unconditional logistic regression (adjusted for sex and age) ORs with 95% CIs. BMI (>25): 1.339 (95% CI: 0.320 to 1.8771); regular physical exercise (yes): 0.576 (95% CI: 0.345 to 2.6751); employed status (yes): 0.976 (95% CI: 0.754 to 1.359); smoking status (no): 0.32 (95% CI:0.716 to 1.271); menstrual cycle phase: (no haemorrhagic phase): 0.893 (95% CI:0.416 to 2.071).

Multinomial logistic regression (adjusted for sex and age) ORs with 95% CI. -Controls vs. mild-moderate RP patients: testosterone: 0.983 (95% CI: 0.722 to 0.895); cortisol: 1.334 (95% CI: 0.964 to 1.582); sAA: 0.83 (95% CI: 0.649 to 1.177); sTNFαRII:0.45 (95% CI: 0.264 to 0.892); sIgA: 1.0 (95% CI: 0.867 to 1.459); melatonin: 0.23 (95% CI: 0.164 to 0.792)-Controls vs. severe RP patients: testosterone: 0.878 (95% CI: 0.692 to 0.990); cortisol: 1.786 (95% CI: 0.764 to 2.312); sAA: 0.89 (95% CI: 0.789 to 1.678); sTNFαRII:0.39 (95% CI: 0.234 to 0.872); sIgA: 1.0 (95% CI: 0.967 to 2.359); melatonin: 0.34 (95% CI: 0.156 to 0.692).

A linear regression analysis aimed at understanding the factors contributing to stress levels with an R-squared value of 0.459 was used to evaluate the capability of salivary biomarkers and disease severity to estimate changes in stress levels in patients with RP. From all salivary biomarkers analyzed in the study, Cortisol (p<0.001) and Melatonin (p = 0.047) were the two best predictors.

**Fig 3:**
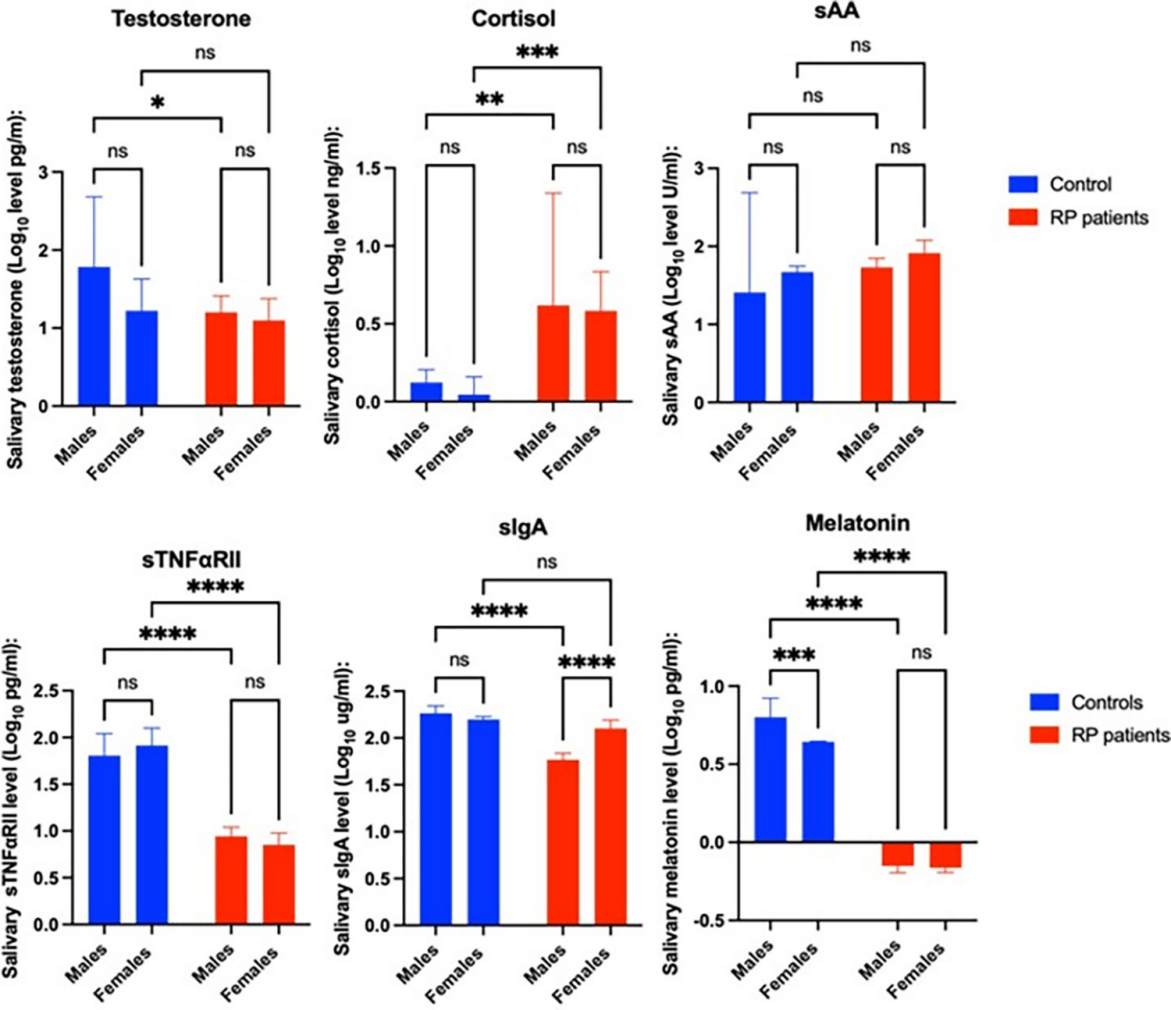
Mean ± SD Log-transformed sSSBC and differences between groups according to sex. ANOVA results.

Before age adjustment, Mean salivary cortisol (0.617 ± 0.721 Log_10_ pg/ml) (p < 0.01), (0.582 ± 0.252 Log_10_ pg/ml) (p < 0.001); sTNFαRII (0.942 ± 0.328 Log_10_ pg/ml) (p < 0.001), (0.852 ± 0.329 Log_10_ pg/ml) (p < 0.001); and melatonin (-0.1510 ± 0.144 Log_10_ pg/ml) (p < 0.001), (-0.1614 ± 0.084 Log_10_ pg/ml) (p < 0.001) concentrations were significantly different in control than in RP male and female patients (Fig 3). Also, significant differences were seen in Mean salivary testosterone and sIgA concentrations in RP males (1.200 ± 0.718 Log_10_ pg/ml), (1.767 ± 0.238 Log_10_ pg/ml), and control males (1.784 ± 1.334 Log_10_ pg/ml) (2.263 ± 0.1184 Log_10_ pg/ml) (p < 0.05) (p < 0.001) (Fig 3). No differences were found between males and females in RP patients, except in sIgA concentrations (1.767 ± 0.238 Log_10_ ug/ml) vs (2.101 ± 0.240 Log_10_ ug/ml) (p < 0.001) (Fig 3). Also, after correction for age, no significant differences in sSSBC between male and female RP patients were found (p = 0.256). Moreover, no correlation between age and sSSBC-Log was found in our sample group of RP patients (testosterone: p = 0.056; r = -0.257); (cortisol:p = 0.578; r = -0.065); (sAA: p = 0.477; r = 0.083); (sTNFαRII: p = 0.071; r = 0.258); (sIgA: p = 0.071; r = -0.068); and (melatonin: p = 0.987; r = -2.315).

**Fig 4:**
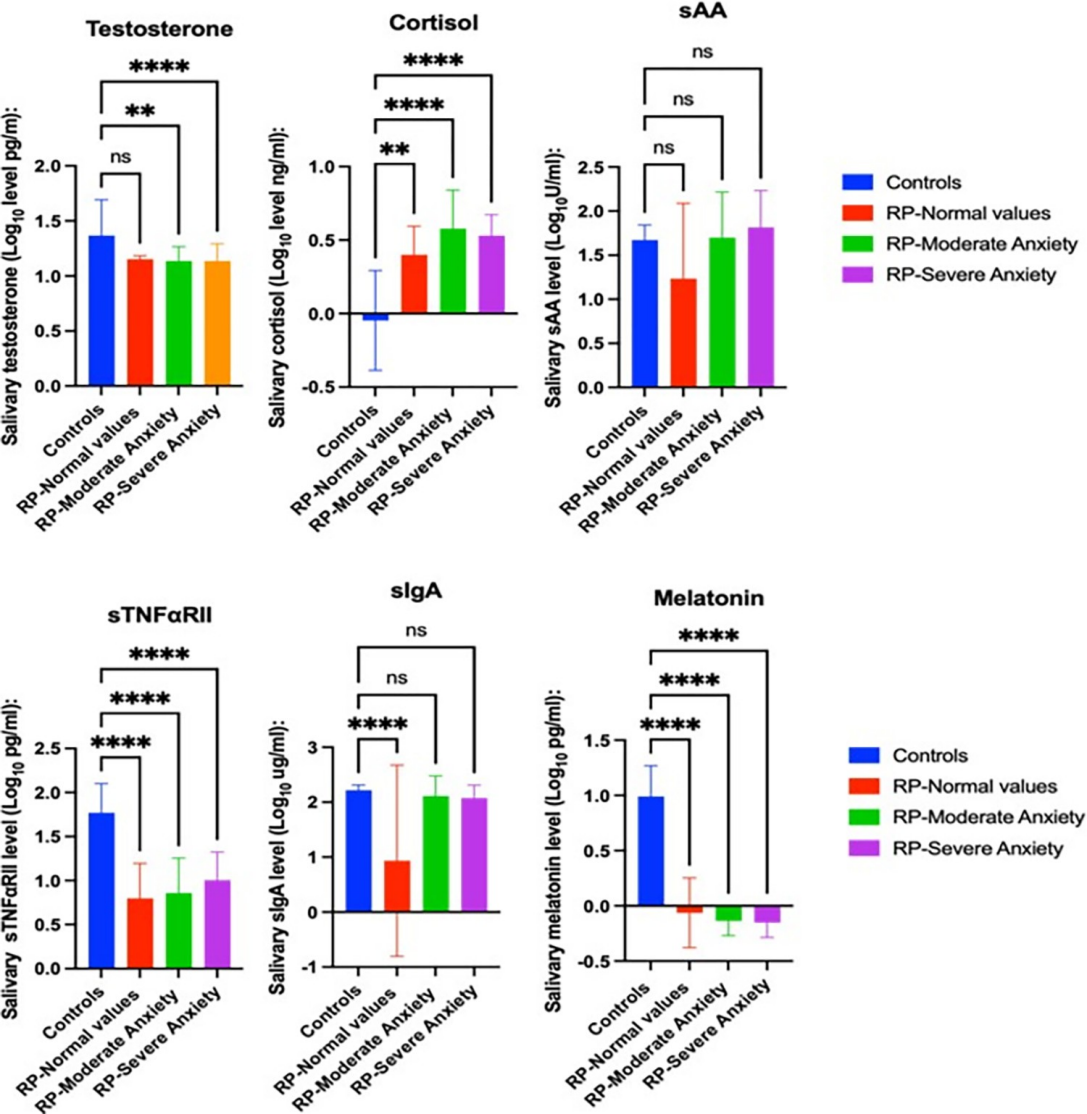
Mean ± SD Log-transformed sSSBC and differences between groups according to anxiety status. ANOVA results.

Mean salivary cortisol (0.400± 0.194 Log_10_ pg/ml) (p < 0.05), (0.5776 ± 0.262 Log_10_ pg/ml) (p < 0.001), (0.530 ± 0.142 Log_10_ pg/ml) (p < 0.001); sTNFαRII (1.234 ± 0.854 Log_10_ pg/ml) (p < 0.001), (1.699 ± 0.516 Log10 pg/ml) (p < 0.001), (1.815 ± 0.416 Log_10_ pg/ml) (p < 0.001); melatonin (0.799 ± 0.394 Log_10_ pg/ml) (p < 0.001), (0.857 ± 0.396 Log_10_ pg/ml) (p < 0.001), (1.005 ± 0.319 Log_10_ pg/ml) (p < 0.001) concentrations were also significantly different in control than in RP patients with a normal, moderate and severe anxiety levels (Fig 4). Mean salivary testosterone was (1.136 ± 0.130 Log_10_ pg/ml) (p < 0.001), (1.135 ± 0.1573 Log_10_ pg/ml) (p < 0.001) significantly different in RP patients with a moderate and severe levels of anxiety as compared to controls (Fig 4). Also, a significant difference in sIgA was found between RP patients with normal values and controls (0.933 ± 1.74 Log_10_ pg/ml) vs. (2.218 ± 0.094 Log_10_ pg/ml) (p < 0.001). No differences were found in sAA levels between groups.

Median salivary sTNFαRII (1.021 ± 0.310 Log_10_ pg/ml) vs. (0.8571 ± 0.321 Log10 pg/ml) (p < 0.05) was significantly different between normal and poor sleepers., with no other differences in the rest of sSSBC values. In addition, no differences were found in lifestyle confounders (smoking status, employment status, BMI, and physical exercise) or potential effect of the female cycle (p > 0.05).

## Discussion

The present study was an evaluation of the usefulness of stress and sleep deprivation-related salivary biomarkers in patients with RP. Most of our results in patients with RP significantly differed from controls, and, in general, the more advanced the disease stage and the worse the results of the anxiety and sleep questionnaires were in the patients’ group, the greater the differences.

The vast majority of RP patients (58.97%) were classified as severely anxious and poor sleepers (58.98%), being more prone to mental disorders such as generalized anxiety disorder or major depression. In this sense, other authors have investigated this association in RP patients; Sakamoto et al. used scores, finding 37% of anxious RP patients and 15.5% of depressed ones; meanwhile, Zhang et al. using the international disease classification (IDC)-10, obtained anxiety diagnosis in 17.78% of patients and depression in 18,70% [7,38]. Moreover, they found a correlation between depression and anxiety incidence in RP patients and their functionality, understood as their visual ability, general health, social function, dependency, etc.[7,39]. These results are in line with the hypothesis that RP could seriously interfere with their everyday lives and that these comorbidities are not rare among RP patients.

Regarding daytime sleepiness, it was higher in RP patients than in control subjects (44.87% of mild-moderate sleepiness in patients vs none in the control group; p>0.001). Our results are according to previous reports. Ionescu et al. concluded that RP patients had increased daytime sleepiness, difficulties in alert response and more sleeping disturbances, their sample was different though since all their 12 patients were legally blind [39]. Otherwise, Ionescu et al. did not find a significant difference in self-reported depression scores between control and RP patients. Therefore, our patients were more anxious and seemed to sleep worse than the controls. In this situation, salivary stress biomarkers results were analyzed. Salivary cortisol was statistically significantly higher in patients with RP than control group and in all patients regardless of their level of anxiety. It has been reported that there is an increased salivary concentration of cortisol related to psychological and physical stress since it correlates well with serum adrenocorticotrophin and reflects the activity in the HPA axis [40]. This same rise has been demonstrated in patients with the post-traumatic disorder, anticipatory stress, depression, or major schizophrenia [19,22,40–42], as well as its relationship with physical stress in patients with cardiovascular stress and cardiometabolic risk [43] or obstructive sleep apnoea syndrome [44,45]. No differences were found between groups of disease severity in salivary cortisol concentration. Meanwhile, sAA, which reflects the activity of SAS in acute stress [40], even if it was higher in patients than controls, this difference was only statistically significant when comparing controls with mild-moderate and severe groups of RP separately.

Testosterone, sTNFαRII and melatonin were significantly higher in the control group than in the patients’ group. Salivary testosterone was reduced in RP patients, probably related to a higher cortisol secretion being the inhibition of testosterone one of the effects that cortisol has on the human body [46]. Therefore, an inverse relationship exists between cortisol and testosterone, which could become even more obvious during acute stress times, with cortisol peaks [47,48]. Testosterone is a hormone that has been correlated to a higher stress perception and more assertive behaviour, but there is not much evidence of its effect on mood, although exogenous testosterone has been proposed as an antidepressant, [49,50]. However, comparing different disease stages, no differences were found between groups, just the control group differed from both groups separately.

Melatonin also seemed to be reduced in RP patients regardless of their level of anxiety. Conversely, no differences were found in melatonin concentration between mild-moderate and severe RP patients. The pineal gland is responsible for melatonin production using photic activation through intrinsically photosensitive retinal ganglion cells (ip-RGCs) that mediate non-visual light responses using melanopsin as photopigment [51,52]. Light information and the light-dark cycle coordinate the production of melatonin in turn, by sympathetic activation in a circadian manner [26,53,54]. Ip-RCGs are also involved in other non-visual functions such as pupil size control and receiving afferents from photoreceptors, especially, in short-duration light exposure [51,52]. Given this situation it is not unlikely that RP patients, as the disease progresses, could suffer from melatonin secretion dysregulation, explaining the differences between patients and controls in salivary melatonin. This relationship has already been described in relation to other ocular diseases, for example, circadian rhythms and sleep patterns are dysregulated in some patients suffering from diabetic retinopathy because of the damage at the ip-RGC level and pupillometric measurements can be altered in glaucoma, RP patients or Leber’s optic neuropathy [26,27,54,55]. This is thought to occur at very late stages, especially if the disease mechanisms start at the outer retina. Nowadays there is no consensus on this issue since some studies have been inconclusive [56,57].

Finally, at the tissue level, melatonin and circadian rhythms have been related to phagocytic functions and antioxidant properties in some ocular structures, such as RPE, photoreceptors, RCGs, and even corneal epithelial cells apart from its relationship with intraocular pressure regulation and aqueous humour production [52]. For example, RPE cells were studied at a preclinical assay conducted by Shakhmantsir et al. on Prpf8 knock-in mice carrying a missense mutation responsible for RP in some patients. They concluded that circadian dysregulation could also contribute to retinal damage together with other push factors in the disease pathogenesis [58]. Turning to our study, circulating solubilized TNF-αRII was used as a biomarker of cellular inflammation due to its relationship with intracellular adhesion molecules (ICAM) and other inflammatory factors [59]. Its concentration in the control group was surprisingly higher than patients’ not being directly related to cortisol in terms of functions. In addition, an inflammatory status in the human body could lead to an endogenous hypercortisolism, with its immunosuppressive effects and potentially changing cytokines and immune system profile. Salivary interleukin-6 (IL-6) [23,60], Interferon-γ (IFN- γ)/Il-4 ratio [61], reactive protein-C [60], IL-1β or myeloperoxidase [23,62] and free light chains [20,63] are also biomarkers that could shed some light and be useful in anxiety and sleep deprivation assessment and should be considered in further studies.

Our linear regression model highlights cortisol and melatonin’s significant correlation with stress levels alongside interesting, though not always significant, trends in other biomarkers with an R-squared value of 0.459. Although this value indicates a moderate relationship between the variables, a significant proportion of the variability remains unexplained, suggesting the possible existence of other factors not considered in the current model.

These findings underscore the complexity of stress as a multifaceted condition influenced by various biological factors. Future research should delve deeper into these relationships, potentially incorporating additional variables to enhance the model’s explanatory power.

This study intended to bring to light the behaviour of salivary stress biomarkers in patients with RP, a chronic, progressing, and disabling disease with severe consequences in everyday life. Certainly, our study had some limitations in addition to its retrospective nature. Fifteen patients had to be excluded from the analysis to avoid bias and most of our patients were classified as having severe or moderate disease severity, not being possible to study the initial stages of the disease. It would be of great interest if it was possible to extend the sample size and study in depth the inflammatory profile of the disease.

In conclusion, sleep disturbances, difficulties in stress coping and anxiety, can have serious and negative consequences for mental and physical health, being RP patients especially vulnerable to them and frequently under-diagnosed. Therefore, sSSBCs are a practical, easy, and non-invasive tool for their assessment and further management, allowing the clinician a holistic approach.
